# PKC-Dependent Human Monocyte Adhesion Requires AMPK and Syk Activation

**DOI:** 10.1371/journal.pone.0040999

**Published:** 2012-07-25

**Authors:** Mei-Ying Chang, Duen-Yi Huang, Feng-Ming Ho, Kuo-Chin Huang, Wan-Wan Lin

**Affiliations:** 1 Department of Pharmacology, College of Medicine, National Taiwan University, Taipei, Taiwan; 2 Department of Internal Medicine, Tao-Yuan General Hospital Department of Health the Executive Yuan, Taoyuan, Taiwan; 3 School of Chinese Medicine, China Medical University, Taichung, Taiwan; 4 Department of Family Medicine, National Taiwan University Hospital Taipei, Taipei, Taiwan; 5 Graduate Institute of Medical Sciences, Taipei Medical University, Taipei, Taiwan; University of Florida, United States of America

## Abstract

PKC plays a pivotal role in mediating monocyte adhesion; however, the underlying mechanisms of PKC-mediated cell adhesion are still unclear. In this study, we elucidated the signaling network of phorbol ester PMA-stimulated human monocyte adhesion. Our results with pharmacological inhibitors suggested the involvement of AMPK, Syk, Src and ERK in PKC-dependent adhesion of THP-1 monocytes to culture plates. Biochemical analysis further confirmed the ability of PMA to activate these kinases, as well as the involvement of AMPK-Syk-Src signaling in this event. Direct protein interaction between AMPK and Syk, which requires the kinase domain of AMPK and linker region of Syk, was observed following PMA stimulation. Notably, we identified Syk as a novel downstream target of AMPK; AICAR can induce Syk phosphorylation at Ser178 and activation of this kinase. However, activation of AMPK alone, either by stimulation with AICAR or by overexpression, is not sufficient to induce monocyte adhesion. Studies further demonstrated that PKC-mediated ERK signaling independent of AMPK activation is also involved in cell adhesion. Moreover, AMPK, Syk, Src and ERK signaling were also required for PMA to induce THP-1 cell adhesion to endothelial cells as well as to induce adhesion response of human primary monocytes. Taken together, we propose a bifurcated kinase signaling pathway involved in PMA-mediated adhesion of monocytes. PKC can activate LKB1/AMPK, leading to phosphorylation and activation of Syk, and subsequent activation of Src and FAK. In addition, PKC-dependent ERK activation induces a coordinated signal for cytoskeleton rearrangement and cell adhesion. For the first time we demonstrate Syk as a novel substrate target of AMPK, and shed new light on the role of AMPK in monocyte adhesion, in addition to its well identified functions in energy homeostasis.

## Introduction

Spleen tyrosine kinase (Syk) is a non-receptor tyrosine kinase, comprising two N-terminal Src homology 2 (SH2) domains, a linker region, and one kinase domain in its C-terminal region [1]. In last decade, Syk has been widely investigated in association with various immunoreceptors and is demonstrated to play crucial roles in innate and adaptive immunity [2]. In addition, Syk is also involved in the signaling of integrins (such as beta2, beta3 and CD11b) [3]. Signaling of Syk, typically in coordination with Src kinase, leads to activation of PLCgamma and PI3K, which are required for the control of cell adhesion, migration, phagocytosis and aggregation [4]–[6].

Besides the well identified signaling pathway that links Syk indirectly to PKC via PLCgamma, which induces phosphoinositide turnover to generate diacylglycerol for PKC activation, direct activation of PKC by Syk was demonstrated. In FcRI-stimulated mast cells, PKCbetaI and PKCalpha are activated by Syk-mediated tyrosine phosphorylation at Tyr662 and Tyr658, respectively [7]. Conversely, some studies have revealed a pathway where Syk is a downstream signal of PKC. Incubation of the purified kinase domain of Syk with PKC demonstrates the ability of PKC isoforms to phosphorylate Syk and enhance its tyrosine kinase activity [8]. Most recently, a study in endothelial cells indicated that PKCdelta-mediated activation of Syk plays an important role in thrombin signaling of NF-kappaB activation and intercellular adhesion molecule-1 expression [9]. Thus, there is a complex signaling interplay between PKC and Syk, which is dependent on cell type and the context of stimulation.

AMP-activated protein kinase (AMPK) is a heterotrimeric serine/threonine kinase composed of a catalytic alpha subunit and regulatory beta and gamma subunits [10]. AMPK activity is absolutely dependent on its phosphorylation at a major activating site (Thr172) of the alpha-subunit by LKB1 and CaMKKbeta. It has been demonstrated that AMPK functions as an intracellular energy sensor that is activated when cells experience energy-depleting stresses [11], [12]. Upon activation, AMPK phosphorylates and inactivates several key enzymes in energy-consuming biosynthetic pathways, while increasing glucose transport, fatty acid oxidation, and glycolysis, thereby stimulating alternative pathways for ATP regeneration. In addition to its role in metabolic processes, AMPK is also implicated as an anti-inflammatory target [13], [14]. Most studies have focused on the role of AMPK in regulating inflammatory gene expression, whereas the possibility of direct regulation of leukocyte adhesion has not been fully examined.

PKC plays a pivotal role in mediating monocyte adhesion; however, the downstream mechanisms mediating its function are not fully elucidated. Thus, in this study, using phorbol 12-myristate 13-acetate (PMA)-stimulated human monocytic leukemia cell line THP-1 as a model system in most experiments, we investigated the signaling network among PKC, Syk and AMPK, and explored their functional relevance in monocyte adhesion.

## Results

### PMA-induced THP-1 monocyte adhesion involves AMPK, Syk and Src

**Figure 1:**
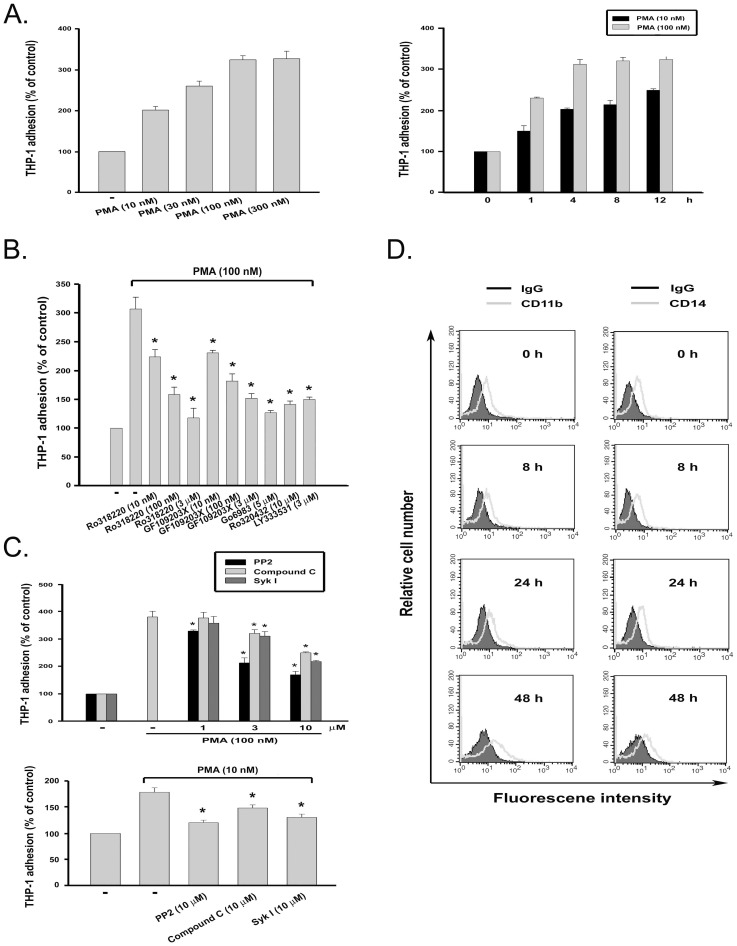
PMA-induced monocyte adhesion is blocked by inhibitors of AMPK, Syk and c-Src. (**A**) THP-1 cells were incubated with PMA at the concentrations indicated for 4 h (left panel) or for different periods (right panel). (**B**) Cells were treated with Ro318220, GF109203X, Go6983, Ro320432, or LY333531 at the concentrations indicated for 30 min prior to the addition of PMA and then incubated for another 4 h. (**C**) All inhibitors at the concentrations indicated were used to pretreat cells 30 min before PMA (10 or 100 nM) incubation for 4 h. Cell adherence was determined by crystal violet staining and quantified by measuring the absorbance at 550 nm. *p<0.05 as compared to the PMA response with vehicle treatment. (**D**) After PMA (100 nM) treatment for different periods, THP-1 cells were stained with control IgG conjugated with fluorochrome, FITC-conjugated CD11b, or PE-conjugated CD14 antibody for flow cytometry.

Previous reports have demonstrated that human monocytic THP-1 leukemia cells can be induced to differentiate along the monocytic lineage following exposure to PMA, a potent tumor promoter capable of activating conventional and novel PKC isoforms. PMA treatment resulted in adherence, loss of proliferation, phagocytosis of latex beads, and expression of CD11b and CD14 [15]. We found that PMA (100 nM) can enhance THP-1 cell adhesion in time- and concentration-dependent manners (Fig. 1A). To elucidate which PKC isoforms are involved in PMA-induced cell adhesion, selective inhibitors of PKCalpha (Ro320432), PKCbeta (LY333531), or nonselective PKC inhibitors (Ro318220, GF109203X and Go6983) were tested for their abilities to block this effect. PMA-triggered cell adhesion within 4 h was reduced in the presence of all tested inhibitors, indicating that PMA-enhanced cell adhesion is via activation of PKCalpha and PKCbeta (Fig. 1B). Furthermore, results revealed that inhibitors of AMPK (compound C), Syk (Syk inhibitor I) and c-Src (PP2) also exerted a concentration-dependent inhibition of PMA response at 100 nM (Fig. 1C, upper panel). Likewise, the induction of adhesion at a lower concentration of PMA (10 nM) was also attenuated by these inhibitors (Fig. 1C, lower panel). To understand if the attenuated responses are resulting from the reduced cell viability, the MTT [3-(4,5-cimethylthizazol-2-yl)-2,5-diphenyl tetrazolium bromide] assay, an index of mitochondrial activity, was performed to address this question. We found that these inhibitors did not affect cell viability after 24 h incubation (data not shown). To understand whether reduced cell adhesion is associated with changes of cell differentiation status, CD11b and CD14 expressions were examined by flow cytometry. As shown in Fig. 1D, only the CD11b expression was increased by PMA (100 nM) treatment for 48 h. These results suggest that the inhibition of cell adhesion by kinase inhibitors within the 4-h incubation period is not attributed to attenuated differentiation of THP-1 cells into macrophages.

**Figure 2:**
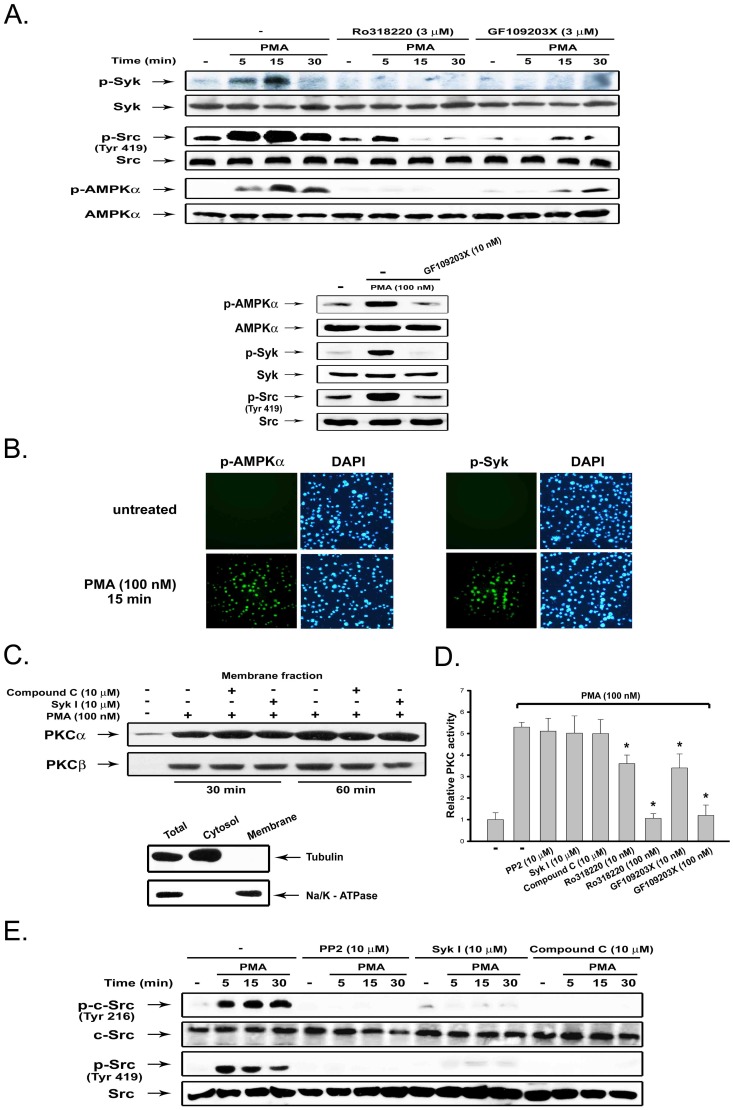
PKC-dependent AMPK and Syk activation mediate Src activation. (**A**) THP-1 cells were incubated with PMA (100 nM) and the indicated PKC inhibitors for various periods, followed by immunobloting. (**B**) THP-1 cells were treated with PMA for 15 min; phosphorylation of AMPK and Syk were analyzed by immunofluorescence staining. DAPI staining was used to determine nuclear localization. (**C**) After drug treatment as indicated, membrane fractions were harvested for immunoblotting of PKCalpha and PKCbeta. To ensure no contamination of fractionation, tubulin and Na/K-ATPase were used as cytosol and membrane controls, respectively. (**D**) PKC activity was determined as described in Methods. *p<0.05 as compared to the PMA response with vehicle treatment. (E) THP-1 cells were pretreated with inhibitors for 30 min, followed by treatment with PMA (100 nM) for the indicated time intervals, and then immunoblotting of protein phosphorylation on Src (Tyr 419) and c-Src (Tyr 216).

To further identify the downstream signaling elements, we analyzed the phosphorylation status of Syk, Src and AMPK. Phosphorylation of human Syk at Y525/526 (equivalent to mouse Y519/520), Src at Y419 (equivalent to mouse Y418 and chicken Src Y416) and AMPKalpha at T172 are activation indexes of the respective kinases. Results shown in Fig. 2A revealed that activation of these kinases, as assessed by their active phosphorylation, was rapidly evoked upon PMA (100 nM) treatment. Meanwhile, pan-PKC inhibitors (Ro318220 and GF109203X) abrogated these effects of PMA at 3 µM (upper panel). Lower concentration of GF109203X (10 nM) exerted a similar effect (lower panel). The ability of PMA to activate AMPK and Syk was further demonstrated by immunofluorescence detection of phosphorylated forms of these proteins (Fig. 2B). These results suggest that AMPK, Syk, and Src are downstream targets of PKC.

Next we examined whether inhibition of PKC-dependent adhesion by pharmacological inhibitors is due to off-target effects. Since targeting PKC to the plasma membrane is a prerequisite for activation, we examined the membrane translocation of PKCalpha and PKCbeta after PMA stimulation. As shown in Fig. 2C, neither compound C nor Syk inhibitor I affected the translocation of PKCalpha and PKCbeta, suggesting the specificities of the kinase inhibitors used. Furthermore, compound C, Syk I, and PP2 did not alter the PMA-induced PKC activity based on the in vitro enzymatic assay. In contrast, PKC inhibitors (Ro318220 and GF109203X) displayed a concentration-dependent inhibition of this response (Fig. 2D).

After observing Syk, Src and AMPK activation are downstream events of PKC, we investigated the order of the signaling cascade. We found that PMA-induced phosphorylation of Src at Tyr419 was dramatically inhibited by PP2, Syk inhibitor I and compound C. Consistent with this finding, results using antibody against Tyr216 phosphorylation on c-Src, which is another index of c-Src activation, also exhibited the same phenomena (Fig. 2E). These data suggest that both AMPK and Syk are upstream signaling molecules mediating Src activation in response to PKC activation.

### A new cascade of PKC-LKB1-AMPK-Syk-Src signaling in monocyte adhesion

**Figure 3:**
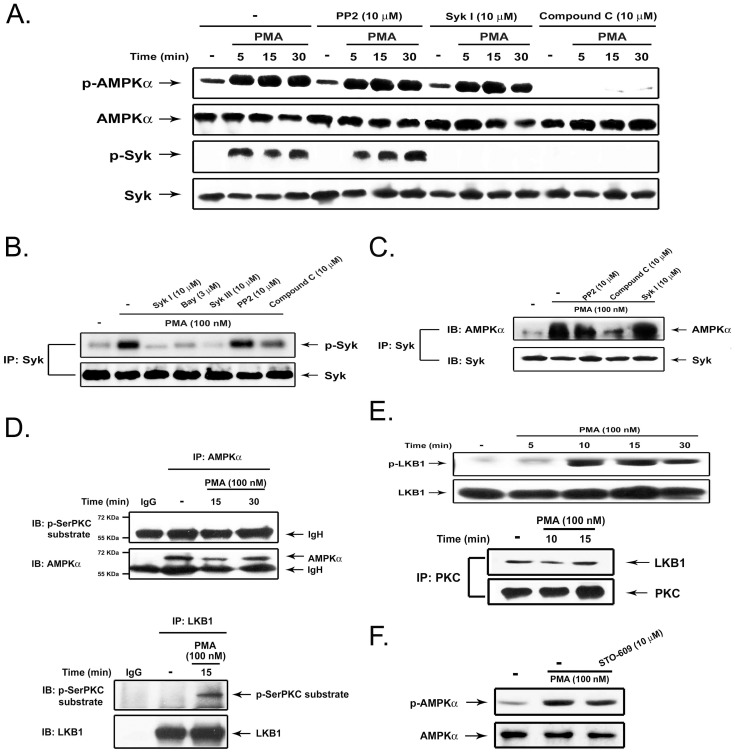
PKC induces the LKB1-AMPK-Syk signal pathway. (**A**) THP-1 cells were treated with inhibitors for 30 min, followed by PMA (100 nM) for the indicated time periods. Immunoblotting using antibodies against AMPK and Syk were conducted. (**B–C**) After pretreatment with inhibitor for 30 min and PMA treatment for 10 min, cell lysates were immunoprecipitated with Syk antibody. Syk enzymatic activity was determined by autophosphorylation (**B**) and AMPK association with Syk was determined by immunoblotting (**C**). (**D**) After PMA treatment, immunoprecipitation with control IgG, anti-AMPKalpha (upper panel) or anti-LKB1 (lower panel) antibody was subjected to immunoblot analysis with anti-phospho-serine PKC substrate antibody. (**E**) Immunoblotting with antibodies against phospho-LKB1 and total LKB1 were conducted (upper panel). In some experiments, PKC immunoprecipitates were subjected to immunobloting with LKB antibody (lower panel). (**F**) THP-1 cells were treated with STO-609 (10 µM) for 30 min, followed by PMA (100 nM) for 30 min. AMPK expression was determined by immunobloting.

Continuing to use pharmacological tools to dissect the relationship between AMPK, Syk and Src, we found that PMA-induced AMPK activation was inhibited by compound C, but not by PP2 and Syk inhibitor I. In contrast, PMA-induced Syk activation was inhibited by Syk inhibitor I and compound C, but not by PP2 (Fig. 3A). In vitro kinase activity assay confirmed the abilities of Syk inhibitors (Syk inhibitor I, Bay 61-3606 and Syk inhibitor III) to block PMA-induced Syk autophosphorylation. Compound C partially decreased this effect, while PP2 failed to affect this event (Fig. 3B). These results suggest that PKC-dependent AMPK activation accounts for Syk, and subsequently Src, activation.

To verify the finding that AMPK is responsible for Syk activation, we examined the protein interaction between both molecules. Results indicate that after PMA stimulation, Syk can associate with AMPK, and this event was reduced by treatment with compound C, while unaffected by PP2 or Syk inhibitor I (Fig. 3C). These results indicate the requirement of kinase activity of AMPK for Syk activation.

Given that PKC mediates AMPK activation, we next explored the molecular mechanism underlying this event. To understand whether AMPK is a direct substrate of PKC, AMPK was immunoprecipitated, then probed with anti-phosphoserine PKC substrate Ab. Results revealed that there is no specific signal detected by anti-phosphoserine PKC substrate in PMA-stimulated AMPK, suggesting that AMPK is not the substrate for PKC (Fig. 3D, upper panel). Next, we investigated if PKC can activate upstream activating kinase LKB1. A previous study demonstrated that PKCzeta can regulate AMPK activity indirectly via activation and phosphorylation of LKB1 at Ser428 [16]. Data obtained from immunobloting with anti-phosphoserine PKC substrate Ab indicated that PMA treatment results in phosphorylation of LKB1 (Fig. 3D, lower panel). Other evidence to support this notion is the increased LKB1 phosphorylation at Ser428 after PMA stimulation (Fig. 3E, upper panel). To confirm this finding, we conducted co-precipitation experiments and found that PKC can bind to LKB1. Notably, their interaction was present in the resting state and was not significantly changed after PMA treatment (Fig. 3E, lower panel). Since CaMKK was reported to mediate AMPK phosphorylation, we also tested this possibility using STO-609, an inhibitor of CaMKK [17]. Results shown in Fig. 3F ruled out this possibility, because AMPK phosphorylation caused by PMA was unaffected by STO-609.

### Syk is phosphorylated and activated by AMPK

**Figure 4:**
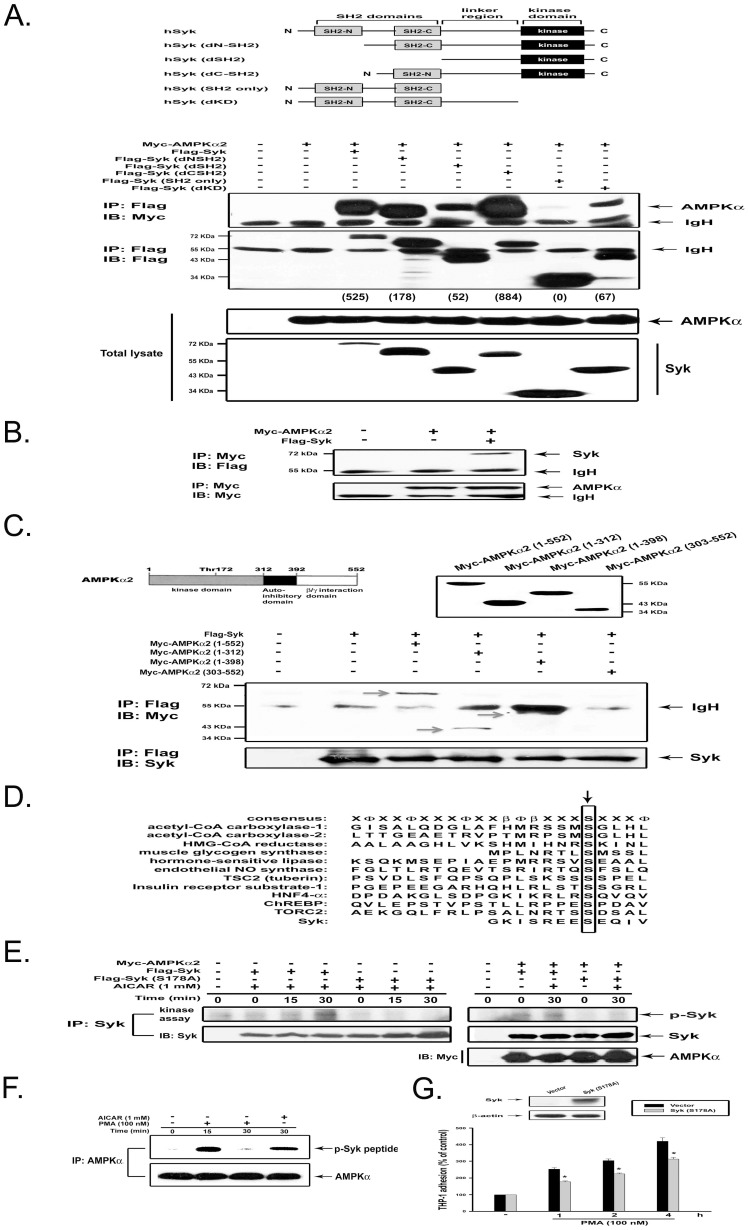
Binding domains between Syk and AMPK and AMPK-mediated Syk phosphorylation. (**A**) HEK293T cells were transfected with Myc-AMPKalpha2 and various deletion constructs of Flag-Syk. Cell lysates were immunoprecipitated with anti-Flag antibody and then subjected to immunoblot analysis with anti-Myc or anti-Flag antibody (upper panel). To assess the association of AMPKalpha2 to different deletions of Syk, we quantified the intensity of immunoprecipitated Syk and associated AMPK. Then the level of AMPK/Syk was calculated (shown in parentheses). Internal controls of Myc-AMPKalpha2 and Flag-Syk expression in cell lysates were determined (lower panel). (**B**) HEK293T cells were transfected with Myc-AMPKalpha2 and Flag-Syk. Immunoprecipitates using Myc antibody were subjected to immunobloting with Flag antibody. (**C**) Plasmids of Flag-Syk and Myc-AMPKalpha2 with various deletions were transfected into HEK293T cells. Immunoprecipitates with anti-Flag antibody were subjected to immunoblot analysis with anti-Myc and -Syk antibodies. (**D**) Alignment of the consensus recognition motifs for AMPK and sequences around sites to be phosphorylated by AMPK on physiological substrates. The serine residues phosphorylated were indicated by the arrow. In the consensus recognition motif, Φ refers to a hydrophobic residue and beta to a basic residue. (**E**) Myc-AMPKalpha2 was expressed with Flag-Syk (WT) or Flag-Syk (S178A) construct in HEK293T. Afterwards, cells were treated with AICAR for 15 or 30 min. Immunoprecipitate with Syk antibody was then subjected to *in vitro* kinase assay. In some samples, the presence of Myc-AMPKα2 in immunocomplexes was determined by immunobloting with Myc antibody. (**F**) THP-1 cells were treated as indicated, and immunocomplexes with AMPK antibody were evaluated in the kinase assay using synthetic Syk peptide containing S178 as a substrate. (**G**) THP-1 cells were transfected with vector or Syk (S178A) plasmids for 48 h, followed by PMA treatment and adhesion assay. *p<0.05 as compared to the control PMA response.

After observing the association of Syk and AMPK under conditions of PKC activation, we would like to map their respective interaction domains. To this end, we made various Flag-tagged DNA constructs of Syk (WT, dNSH2, dCSH2, dSH2, dKD and SH2 only) (Fig. 4A, upper panel). Next, we compared the binding of these Syk molecules to AMPKalpha2 in HEK293T cells, which do not express endogenous Syk. After immunoprecipitation of Syk with anti-Flag antibody and detection of AMPKalpha2 in the immunoprecipitate with anti-Myc antibody, we found that all deleted Syk proteins except Syk (SH2 only) can interact with AMPKalpha2 (Fig. 4A, lower panel). These results suggest that the linker region of Syk is required for interaction with AMPKalpha2. The association of Syk and AMPK was confirmed by reverse immunoprecipitation with anti-Myc antibody (Fig. 4B). Conversely, in order to define the domain of AMPK necessary for the association with Syk, we tested four Myc-tagged AMPKalpha2 constructs (Fig. 4C, upper panel). After immunoprecipitation of Flag-Syk (WT), we detected the presence of AMPKalpha2 (1–552), AMPKalpha2 (1–312), and AMPKalpha2 (1–398), but not AMPKalpha2 (303–552) in the immunoprecipitates (Fig. 4C, lower panel). These results suggest that the kinase domain of AMPK is necessary for the association with Syk.

Next, we tested whether AMPK interaction with Syk can induce Syk phosphorylation and activation. After alignment of the consensus phosphorylation motif for AMPK and confirmation using variant synthetic peptides substrates [18], [19], we predicted Syk might be phosphorylated by AMPK at Ser178 (Fig. 4D). With the use of site-directed mutagenesis, Ser178 of Syk was mutated into alanine (S178A) to determine its activation status versus wild-type Syk by AMPK. HEK293T cells were transiently transfected with the expression vectors for Flag-Syk (WT) or Flag-Syk (S178A) with AMPKalpha2, then stimulated with AICAR. Flag-Syk (WT) and Flag-Syk (S178A) immunoprecipitated with Syk antibody were tested for enzyme activity in an *in vitro* autophosphorylation assay. As shown in Fig. 4E, AICAR indeed activated Syk at 15 and 30 min treatment, while Syk mutant (S178A) was not activated under the same conditions. Moreover, overexpression of AMPK also caused Syk activation in the absence of AICAR, and this effect was not detected in cells expressing mutant Syk. To confirm this finding, a Syk peptide (CWFHGKISREESEQIV) containing the amino acid sequences of Syk at 167–182 was synthesized as substrate in an *in vitro* kinase assay. We found that AICAR- and PMA-activated AMPK indeed caused Syk peptide phosphorylation (Fig. 4F). Moreover, expression of Syk (S178A) reduced cell adhesion in response to PMA (Fig. 4G), confirming Syk is a downstream substrate of AMPK.

### AMPK activation itself is not sufficient for monocyte adhesion but increases the adhesive response to PMA

**Figure 5:**
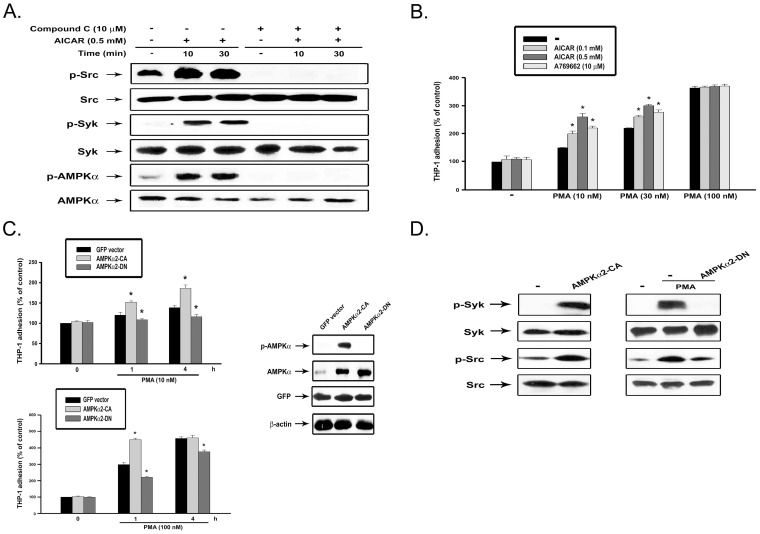
AICAR induces Syk and c-Src activation, and enhances cell adhesion upon PMA stimulation. (**A**) After treatment with agents as indicated, cell lysates were subjected to immunoblotting. (**B**) THP-1 cells were co-incubated with AICAR (0.1, 0.5 mM), A769662 (10 µM) and/or PMA (10–100 nM) for 4 h, followed by cell adhesion assay. (**C**) After expression of active or kinase dead AMPK, THP-1 cells were harvested and tested for the PMA (10, 100 nM)-induced cell adhesion. The expression and activity of AMPK were determined by immunobloting. *p<0.05 as compared to the PMA response without AICAR, A769662, or AMPK adenovirus infection. (**D**) THP-1 cells were infected with adenovirus expressing constitutively active or kinase-dead AMPK. After 48 h infection, cells were either stimulated with PMA (100 nM) or not for 30 min, and Syk and Src activities were determined by immunoblotting.

The above findings suggest the crucial role of AMPK in PKC-mediated monocyte adhesion. We explored if AMPK activation itself was sufficient to induce cell adhesion. To this end, we tested the effects of AICAR, an AMPK activator, on monocytes in terms of signaling-associated cell function. As shown in Fig. 5A, AICAR treatment can rapidly induce AMPK, Syk and Src activation, and all these events were abrogated by compound C. Notably, treatment with AICAR (0.1 and 0.5 mM) or A769662 (10 µM) alone, two AMPK activators [20], did not affect monocyte adhesion, however they did increase cell adhesion elicited by PMA at 10 and 30 nM, but not that at 100 nM (Fig. 5B). The adhesion of total THP-1 cell suspension was fully achieved by PMA (100 nM) stimulation for 4 h, therefore there was not potential for further increase of cell adhesion upon adding AICAR. These results suggest that activation of AMPK by AICAR can enhance the adhesive response. To confirm the results observed with AICAR treatment, we employed a genetic approach. Even though THP-1 cells constitutively expressing active or kinase-dead AMPKalpha2 exhibited the same extent of cell adhesion in the resting state, the two genetic manipulations had opposite effects on the adhesive response to PMA (Fig. 5C). PMA (10 or 100 nM)-induced cell adhesion at 1 and 4 h was inhibited by kinase-dead AMPK. In contrast, cells with active AMPK responded to PMA (10 or 100 nM) more rapidly, and reached the maximal adhesive effect upon treatment with higher concentration of PMA (100 nM) for 1 h. These results suggest that AMPK activity is a modulator of PKC-dependent monocyte adhesion, but activation alone is not sufficient for this event. Furthermore, overexpression of active AMPK leads to the increased phosphorylation of Syk and Src, while kinase-dead AMPK inhibits both tyroinse kinase activations caused by PMA (Fig. 5D). Altogether, these data confirm AMPK as a pivotal mediator for PKC-induced downstream signaling and cell adhesion.

### PKC-dependent ERK activation contributes to monocyte adhesion

**Figure 6:**
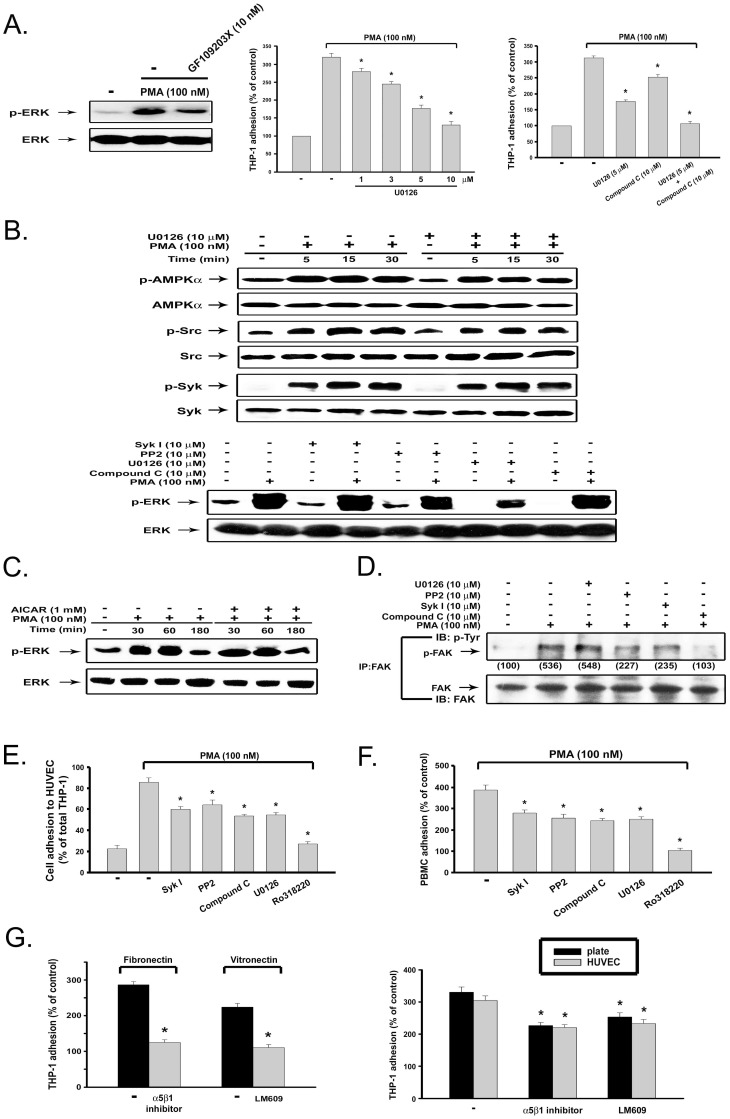
ERK activity independent of AMPK-Syk-Src signaling participates in PKC-mediated monocyte adhesion. (**A**) After pretreatment with 10 nM GF109203X for 30 min, followed by PMA (100 nM) treatment for 10 min, cell lysates were subjected to immunobloting (left panel). In some experiments, after pretreatment with U0126 and/or compound C for 30 min, THP-1 cells were stimulated with PMA (100 nM) for 4 h, and cell adhesion was determined (middle and right panels). (**B–D**) THP-1 cells were treated with PMA in the presence of vehicle or pharmacological agents for 10 min (lower panel of **B**, **D**) or indicated time periods (upper panel of **B**, **C**). Immunobloting with specific antibodies was conducted. FAK activation was determined by immunoprecipitation with FAK-specific antibody and immunoblotting with phospho-tyrosine antibody (**D**). (**E**) THP-1 cells pre-labeled with BCECF were treated with SykI, PP2, compound C, U0126 (each at 10 µM), or Ro318220 (3 µM) for 30 min prior to the addition of PMA (100 nM). After 1 h incubation, THP-1 cells were washed with complete medium twice, and then added to HUVEC monolayer grown in 96-well plate. After 1 h, adhesion of THP-1 cells to HUVEC was determined. (**F**) Human primary monocytes were similarly treated with inhibitors and PMA, and cell adhesion at 4 h was determined. (G) PMA-induced cell adhesion to matrix-coated culture plates, in the absence or presence of alpha5beta1 inhibitor and alphaVbeta3 blocking antibody (LM609), was determined (left panel). In some experiments, after pretreatment with integrin inhibitors for 30 min, THP-1 cells were stimulated with PMA (100 nM) for 4 h, and cell adhesion to either the culture plate or to HUVEC was determined (right panel).

To determine if other signaling events are required to mediate PKC-dependent monocyte adhesion, we tested the role of ERK, a major downstream signaling pathway of PKC, in THP-1 monocyte adhesion. First, PMA-induced ERK phosphorylation was markedly reduced by 10 nM GF109203X (Fig. 6A, left panel). Next we found that U0126 (a specific inhibitor of MEK/ERK) can inhibit PMA-induced THP-1 monocyte adhesion in a concentration-dependent manner (Fig. 6A, middle panel). Co-treatment with UI0126 (5 µM) and compound C (10 µM) achieved a full abrogation of PMA-induced cell adhesion (Fig. 6A, right panel). Despite the ability to block cell adhesion, U0126 did not affect AMPK, Syk or Src activation induced by PMA (Fig. 6B, upper panel). Conversely, PMA-induced ERK activation was not altered by the inhibitors of Syk, Src or AMPK (Fig. 6B, lower panel). Moreover, consistent with our previous findings in rat vascular smooth muscle cells and HUVEC [21], AICAR itself cannot induce ERK activation (data not shown) or affect PMA-induced ERK phosphorylation (Fig. 6C). These results suggest a PKC-activated bifurcated pathway to induce monocyte adhesion through both AMPK-Syk-Src and ERK signaling cascades. Since protein tyrosine kinase focal adhesion kinase (FAK) was described as an index of cell adhesion, we determined its relationship with the two signaling pathways mediated by PKC as proposed. We found that PMA-induced FAK phosphorylation was attenuated by Syk inhibitor I, PP2 and compound C, but was unaffected by U0126 (Fig. 6D). Thus, we suggest that the FAK-mediated cytoskeletal reorganization for PKC-dependent monocyte adhesion is downstream of AMPK-Syk-Src but not the ERK signaling pathway.

Next, we examined whether the protein kinases mentioned above are also involved in THP-1 cell adhesion to HUVEC, as well as the adhesion of primary monocytes to culture plates. To this end, THP-1 cells pre-treated with indicated agents and labeled with fluorescent dye were added to HUVEC for adhesion. We found that SykI, PP2, compound C and U0126 were effective to inhibit the adhesion of THP-1 cells to HUVEC, and Ro318220 can abrogate this response of PMA (Fig. 6E). Similar effects of these kinase inhibitors on PMA-induced adhesion of primary human monocytes to culture plates were observed (Fig. 6F).

Concerned that the adhesion data shown in Fig. 6E was interfered with cytotoxicity of HUVEC, we measured LDH in the culture medium of HUVEC, either in the presence or absence of PMA-stimulated THP-1 for 4 h. As a result, we found no significant difference of LDH level with or without THP-1 adhesion (data not shown). In addition, we also measured TNFalphain the culture medium of THP-1 cells to understand if TNFalpha plays a role for monocyte adhesion to EC. Our data revealed that after PMA stimulation for 4 h (the time for us to measure cell adhesion), TNFalpha concentration (22±8 pg/ml, n = 3) in the medium was not changed. Moreover, in the LDH assay we did not observe significant cytotoxicity in HUVEC treated with TNFα for 4 h (data not shown). These results all together ruled out the intervention of cellular integrity and autocrine factor in our adhesion data.

To understand if integrins might play a role in PMA-induced monocyte adhesion, we examined the effects of alpha5beta1 inhibitor and alphaVbeta3 blocking antibody (LM609), which interfere with the interaction through fibronectin and vitronectin respectively, on PMA-stimulated monocyte adhesion to culture dishes and HUVEC [22], [23]. Before this experiment, we first confirmed the effective concentration (10 µg/ml) of alpha5beta1 inhibitor and LM609 to abrogate fibronectin- and vitronectin-induced cell adhesion (Fig. 6G, left panel). Next, we observed that both integrin inhibitors can reduce PMA-induced THP-1 adhesion to culture plate and HUVEC (Fig. 6G, right panel). These results suggest that integrins are involved in PMA-induced cell adhesion.

## Discussion

In this study, we demonstrate that PKC, Syk, AMPK, Src and ERK kinases play critical roles in monocyte adhesion, and propose a novel signaling mechanism of this event. Previous studies have implicated PKC and Syk in the adhesive functions of integrins [3], [24]. Most findings propose that Syk is the upstream signal to induce PKC activation either indirectly via PLC activation or directly via physical interaction [2], [7], [24]. In contrast, a reverse signaling cascade between PKC and Syk is also demonstrated in endothelial cells where thrombin can induce NF-kappaB activation through PKCdelta-Syk pathway [9]. In this study we unveil a novel signaling network linking PKC activation to downstream Syk via AMPK.

Besides the relatively well identified role of AMPK in metabolic processes, this energy sensor kinase is also implicated as an anti-inflammatory target. AICAR, the pharmacological activator of AMPK, has been reported to exert anti-inflammatory and immunomodulatory effects in various models of inflammation [25]–[27]. In endothelial cells, AMPK activity is associated with phosphorylation and activation of eNOS, resulting in anti-inflammatory action in the vascular wall [28]. Endotoxin lipopolysaccharide-induced expression of proinflammatory molecules and mediators in primary macrophages, microglia, astrocytes, and mesangial cells are suppressed by AICAR [29]–[31]. Mechanistic studies suggest inhibitions of NF-kappaB, c/EBPbeta and reactive oxygen species production contribute to the anti-inflammatory event mediated by AMPK signaling [29], [32]–[36].

Despite the above reports implicating AMPK as an anti-inflammatory molecule, contrary findings of AMPK-dependent inflammatory responses are also documented. In this respect, AICAR and adiponectin can stimulate IL-6 production in fibroblasts via an AMPK-dependent pathway [37]. Moreover, we previously found that AICAR alone can induce cyclooxygenase-2 expression in several types of cells under resting status [21]. We thus postulate that AMPK has a bi-directional function and might act as an “early warning and protective signal” under sub-pathological stress. Cells under inflammatory stress upon LPS or proinflammatory cytokine stimulation would favor AMPK to play a role in resolving inflammation and tissue protection. In contrast, at a resting basal situation, AMPK-dependent upregulation of cyclooxygenase-2 might exert a homeostatic function through eicosanoid production.

Leukocyte adhesion is the essential step required for circulating monocytes to traverse multiple tissue barriers, and to perform their functions in host defense. Both lower expression of proinflammatory cytokines, chemokines, and adhesion molecules, and upregulation of eNOS activity in endothelial cells might contribute to the AMPK-mediated inhibition of leukocyte-endothelial cell interaction [26], [29]–[31], [38]. Currently only one study has shown the direct role of AMPK in regulating leukocyte adhesion [39]. In this study, AMPK was found to mediate rosiglitazone-induced inhibition of fibronectin-mediated adhesion of human peripheral blood mononuclear cells. The difference between this result and ours might be due to ligand (integrin vs direct PKC activator) and cell type specificity.

In this study, we proved that the upstream kinase of AMPK, LKB1, is a substrate of PKC in THP-1 monocytes stimulated by PMA. We ruled out that AMPK has a direct interaction with PKC or involvement of CaMKK in this event. Moreover, we also provide evidence to support that Syk is a novel biological substrate of AMPK, thus extending the functional impact of AMPK in regulation of leukocyte adhesion. First, Syk can directly interact with AMPKalpha2 via the linker region of Syk and the kinase domain of AMPK. Second, the direct interaction between Syk and AMPKalpha2 is induced upon AMPK activation. Third, Syk-AMPKalpha2 interaction leads to AMPK-mediated phosphorylation of Syk at Ser178, which is located at the C terminal region of SH2 domain. Using Syk synthetic peptides as a substrate (CWFHGKISREESEQIV), we further confirmed the phosphorylation site by AMPK at the Ser178 residue of Syk. Fourth, phosphorylated Syk in turn exerts its activation and propagates downstream signals to Src and FAK for cell adhesion. The cross-activation of Syk and Src has been demonstrated to exert the cellular functions of immunoreceptors and integrins [4]. Fifth, using a direct AMPK activator (AICAR) and genetic approach (AMPK-CA and AMPK-DN) we confirmed the role of AMPK in activation of Syk, Src and increase of monocyte adhesion.

It is interesting to note that even though AMPK is required to mediate PKC-dependent monocyte adhesion, AMPK activity alone is not sufficient for this event. In other words, a coordinate signal pathway downstream of PKC and independent of AMPK is required to evoke cell adhesion. In this respect, we identify ERK as the major player in this event. The PKC-mediated AMPK-Syk-Src-FAK pathway proposed above is not affected by ERK inhibitor, while ERK inhibitor can reduce cell adhesion. Moreover, the failure of inhibitors of AMPK, Syk, and Src to affect PMA-induced ERK phosphorylation, and the inability of AICAR to induce ERK activation suggest that a bifurcated signaling pathway is induced and is crucial to PKC-dependent cell adhesion. We propose that to achieve firm leukocyte adhesion, leukocytes need to collect different intracellular signals.

The involvement of ERK in PKC-mediated THP-1 cell adhesion has been documented [22]. Molecular mechanistic studies provide evidence for the role of ERK signaling in adhesion assembly. In this respect, PMA-induced activated ERKs are localized to focal adhesions, and promote Rho-dependent focal adhesion formation by suppressing p190A RhoGAP [40], [41]. Moreover, some actin-binding proteins as phosphorylation targets of ERKs and contributing to focal adhesion formation have been demonstrated [42]–[44]. Indeed our current study using two integrin inhibitors suggests the involvement of integrins, at least alphaVbeta3 and alpha5beta1, in PMA-induced THP-1 monocyte adhesion to either the culture dish without further matrix coating or to HUVEC. The contribution of alphaVbeta3 via ERK activation has been demonstrated to mediate PMA-induced THP-1 adhesion [22]. Further study is required to elucidate the link of the bifurcated signaling pathways downstream of PKC leading to alpha5beta1 activation.

**Figure 7:**
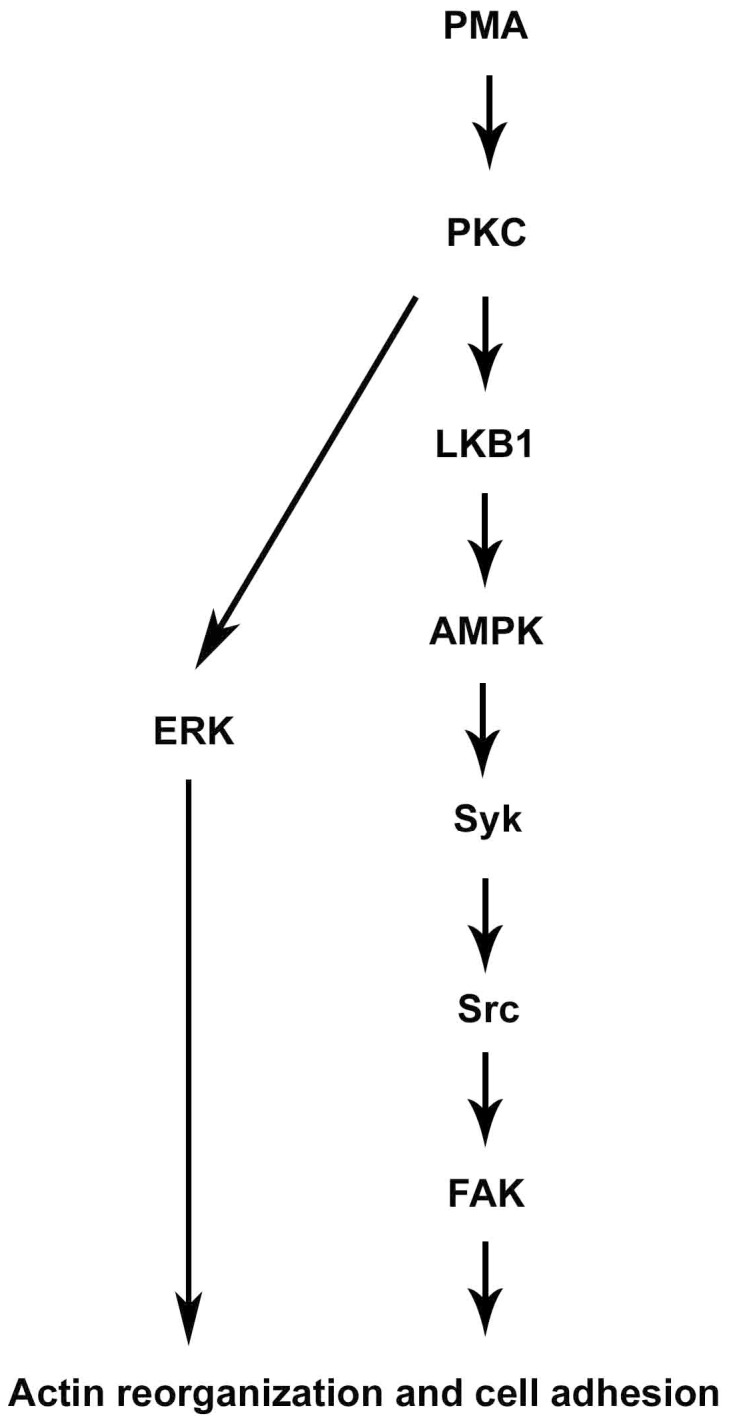
A bifurcated signaling pathway is involved in PMA-mediated adhesion of monocytes. PKC can activate LKB1/AMPK, leading to phosphorylation and activation of Syk, then Src and FAK subsequently. PKC-dependent ERK activation induces a coordinated signal for cytoskeleton rearrangement and cell adhesion.

In summary, our data suggest that a coordinated signaling between AMPK-Syk-Src-FAK and ERK is involved in PMA-induced monocyte adhesion (Fig. 7). We for the first time unveil a novel signaling between AMPK and Syk, which may shed new light on the roles of AMPK in cell adhesion, in addition to its well identified functions in energy homeostasis, insulin sensitivity and obesity.

## Materials and Methods

### Cell Culture and Reagents

THP-1 cells purchased from ATCC were maintained in RPMI 1640 medium containing 10% FBS. HEK293T and 293F cells purchased from ATCC were maintained in DMEM complete medium. Human umbilical vein endothelial cells (HUVEC) were purchased from ScienCell (Carlsbad, CA, USA). Primary monocytes were prepared from the blood of healthy human donors and cultured in DMEM as described previously [45]. Experiments of human samples have been approved by Institutional Review Boards (IRB) of National Taiwan University Hospital, Taipei, Taiwan (201111055RIC). HUVEC were purchased from ScienCell (Carlsbad, CA) and cultured as we previously described [21].

DMEM, RPMI 1640, FBS, penicillin, and streptomycin were obtained from Gibco BRL. The enhanced chemiluminescence detection agents were purchased from Amersham Biosciences. Lipofectamine™ LTX (Cat No. 15338-100) and Plus™ Reagent (Cat. No. 11514-015) were obtained from Invitrogen. Inhibitor of alpha5beta1 and anti-CD11b-FITC were purchased from Santa Cruz Biotechnology. Antibodies specific for AMPKalpha, Syk, ERK and phosphorylated AMPKalpha (Thr172), Syk (Tyr525/526), ERK1/2 (Thr202/Tyr204), GFP, tubulin and Na/K-ATPase were purchased from Cell Signaling. Antibody against human Src (Tyr419, equivalent to Tyr416 of chicken Src and v-Src) was purchased from Invitrogen. Anti-Flag bead antibody (M2-beads), anti-Flag antibody, STO-609 and other chemicals were obtained from Sigma Aldrich. Integrin–alphaVbeta3 blocking antibody (LM609) was obtained from Chemicon (Temecula, CA). Anti-CD14-PE was purchased from Bioscience. All materials for SDS-PAGE were obtained from Bio-Rad. Ro318220, GF109203X, PMA, Compound C, Syk inhibitors (Syk inhibitor I, Bay 61-3606, Syk inhibitor III), PP2, Go6983, Ro320432, A769662 and U0126 were purchased from Calbiochem. LY333531 was purchased from Alexis Biochemicals. Human fibronectin and vitronectin were purchased from R&D systems (Minneapolis, MN). PKC activity kit was purchased from ENZO Life Sciences (Farmingdale, NY).

### Adhesion Assay

THP-1 cells (5×10^4^ cells) and human primary monocytes (2×10^4^ cells) in complete culture medium containing 10% FBS were plated to tissue culture 96-well plate (Costar 3599 wells, Corning Incorporated), and pretreated with the indicated kinase inhibitors for 30 min, followed by stimulation with PMA (1–100 nM) for different periods. Nonadherent cells were removed by gentle washing with PBS before fixation with methanol for 10 min, followed by addition of 0.1% (v/v) crystal violet in PBS for 15 min. After washing with distilled water three times, acetic acid was added to a final concentration of 33% (v/v) to solubilize cell lysates, followed by absorbance measurement at 550 nm. In some experiments to determine the adhesive response to HUVEC, THP-1 cells were pre-labeled with 0.1 µg/ml BCECF-AM for 1 h at 37°C. Then cells were washed and treated with indicated agents including kinase inhibitors and/or PMA for 1 h. Cells were washed twice with growth medium, and equal amount of THP-1 cells were added to EC monolayer cultured in 96-wells for 1 h. After removal of culture medium and non-adherent THP-1 cells, the number of adherent cells was determined by measuring the fluorescence intensity.

### Plasmids

Myc-AMPKalpha2, Myc-AMPKalpha2 (1–312), Myc-AMPKalpha2 (1–398), and Myc-AMPKalpha2 (303–552) were provided from Dr. Kelly A. Wong (Whitehead Institute for Biomedical Research, Cambridge, MA). Flag-tagged wild type human Syk (1–635) was constructed by ligating the PCR-derived DNA fragment spanning the open reading frame region of Syk with the EcoRI/SalI digested pCMV2-FLAG vector. The regions covering 107–635, 260–635 or 1–369 amino acids of hSyk cDNA were amplified by PCR and inserted to EcoRI/SalI digested pCMV2 FLAG to generate the N-SH2-del (dNSH2), SH2-del (dSH2) or kinase domain-del (dKD) of hSyk. The C-SH2-del hSyk (dCSH2) construct was amplified by PCR covering 1–106 and 260–635 amino acids of hSyk. To generate S178A Syk, we used the Quickchange site-directed mutagenesis kit (Stratagene), and serine 178 in the human Syk cloned in the pBlue-script vector was substituted with alanine by changing the codon from ATC to AGC. The mutated primers used were primer 1 (5′-CTC TCG GGA AGA AGC TGA GCA AAT TGT-3′) and primer 2 (5′-ACA ATT TGC TCA GCT TCT TCC CGA GAG) for hSyk (S178A) mutation.

### Cell Transfection and Infection

HEK293T cells (1×10^6^ cells/well) were transfected using lipofectamine™ and Plus™ Reagent with 3 µg plasmid for 4 h, and then changed to complete medium. After 24 h transfection, cells were harvested, followed by collection of cell lysates for immunoprecipitation or western blot. GFP-tagged constitutively active adenovirus-AMPKalpha2-CA and adenovirus-DN kinase-dead (K45R) variant of AMPKalpha2 provided by Dr. Benoit Viollet (INSERM, Paris, France) and Dr. Morris Birnbaum (Univ. Pennsylvania, USA) respectively were prepared in 293F cells and purified by BD-Adeno-X™ Virus Purification kits. THP-1 cells were infected with virus at M.O.I. of a 20∶1 ratio for 48 h.

THP-1 cells (1×10^6^ cells/well) were transfected using lipofectamine™ LTX and Plus™ Reagent with 3 µg plasmid for 4 h, and then changed to complete medium for 48 h. The combination of both reagents can enhance transfection efficiency in THP-1 cells as mentioned by manufacturer. Using pEGFP-C2 expression and fluorescent microscopy as index, we found that the transfection rate in THP-1 cells was 60%.

### Flow Cytometry

THP-1 monocytes (1×10^6^ cells/well) were harvested, fixed with 2% paraformaladehyde, stained extracellularly in 1% FBS/PBS with equal concentrations of control IgG conjugated with fluorochrome (FITC or PE), FITC-conjugated CD11b, or PE-conjugated CD14 antibody for 1 h and then analyzed by flow cytometry.

### Immunoblot and Immunoprecipitation

Immunoblot and immunoprecipitation were performed as described previously by us [21].

### In Vitro Kinase Assay

To determine in vitro kinase activities of Syk, 500 µg of total protein extracts from stimulated cell lysates were pre-cleared for 30 min and then immunoprecipitated with 1 µg anti-Syk antibody overnight, followed by addion of 10 µl protein A/G-agarose beads and rotation at 4°C or another 1 h. The immunocomplexes were washed 3 times with cold lysis buffer (20 mM Tris-HCl, pH 7.5, 150 mM NaCl, 1% Triton X-100, 1 mM MgCl_2_, 2.5 mM beta-glycerophosphate, 5 mM NaF, 100 µM Na_3_VO_4_, 1 mM PMSF, protease inhibitor cocktails) and once with the kinase reaction buffer (20 mM HEPES pH 7.5, 10 mM MgCl_2_, 5 mM *p*-nitrophenyl phosphate). The beads were then incubated for 30 min at 30°C in 20 µl kinase reaction buffer supplemented with 10 µCi of γ-^32^P-ATP. The precipitated complexes were added with 5 µl 5× Laemmli sample buffer and heated at 95°C for 5 min. After centrifuging the samples, the supernatants were run on 10% SDS-PAGE, followed by autoradiography.

### PKC Kinase Activity Assay

THP-1 cells incubated in 6-well plates were pretreated with the indicated kinase inhibitors for 30 min, followed by stimulation with PMA (100 nM) for 1 h. PKC kinase activity was measured using the PKC kinase activity kit according to the manufacturer's instructions.

### Statistical Evaluation

Values are expressed as the mean ± SEM of the least three experiments. ANOVA and Dunnett's tests were used to assess the statistical significance of the differences, with P values of less than 0.05 being considered statistically significant.
